# Prediction of a miRNA‐mRNA functional synergistic network for cervical squamous cell carcinoma

**DOI:** 10.1002/2211-5463.12747

**Published:** 2019-11-17

**Authors:** Dan Sun, Lu Han, Rui Cao, Huali Wang, Jiyong Jiang, Yanjie Deng, Xiaohui Yu

**Affiliations:** ^1^ Obstetrics and Gynecology Dalian Maternity and Child Health Care Hospital China

**Keywords:** cervical intraepithelial neoplasia, cervical squamous cell carcinoma, differentially expressed gene, GEO, miRNA

## Abstract

Cervical squamous cell carcinoma (CSCC) accounts for a significant proportion of cervical cancer; thus, there is a need for novel and noninvasive diagnostic biomarkers for this malignancy. In this study, we performed integrated analysis of a dataset from the Gene Expression Omnibus database to identify differentially expressed genes (DEGs) and differentially expressed miRNAs (DEmiRNAs) between CSCC, cervical intraepithelial neoplasia (CIN) and healthy control subjects. We further established protein‐protein interaction and DEmiRNA‐target gene interaction networks, and performed functional annotation of the target genes of DEmiRNAs. In total, we identified 1375 DEGs and 19 DEmiRNAs in CIN versus normal control, and 2235 DEGs and 33 DEmiRNAs in CSCC versus CIN by integrated analysis. Our protein‐protein interaction network indicates that the common DEGs, *Cyclin B/cyclin‐dependent kinase 1* (*CDK1*), *CCND1*, *ESR1* and *Aurora kinase A* (*AURKA*), are the top four hub genes. P53 and prostate cancer were identified as significantly enriched signaling pathways of common DEGs and DEmiRNA targets, respectively. We validated that expression levels of three DEGs (*TYMS*, *SASH1* and *CDK1*) and one DEmiRNA of hsa‐miR‐99a were altered in blood samples of patients with CSCC. In conclusion, a total of four DEGs (*TYMS*, *SASH1*, *CDK1* and *AURKA*) and two DEmiRNAs (hsa‐miR‐21 and hsa‐miR‐99a) may be involved in the pathogenesis of CIN and the progression of CIN into CSCC. Of these, *TYMS* is predicted to be regulated by hsa‐miR‐99a and *SASH1* to be regulated by hsa‐miR‐21.

## Abstract 2

Here, we performed integrated analysis of a Gene Expression Omnibus database dataset to identify differentially expressed mRNAs and differentially expressed miRNAs in cervical intraepithelial neoplasia (CIN) and cervical squamous cell carcinoma. We identified a total of four differentially expressed genes (*TYMS*, *SASH1*, *Cyclin B*/*cyclin‐dependent kinase 1* and *Aurora kinase A*) and two differentially expressed miRNAs (hsa‐miR‐21 and hsa‐miR‐99a) that may be involved in the pathogenesis of CIN and the progression of CIN into cervical squamous cell carcinoma.

## Graphical abstract

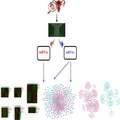

**Abbreviations**
CIN: cervical intraepithelial neoplasia
CSCC: cervical squamous cell carcinoma
DEG: differentially expressed gene
DEmiRNA: differentially expressed miRNA
FDR: false discovery rate
GEO: Gene Expression Omnibus
GO: gene ontology
KEGG: Kyoto Encyclopedia of Genes and Genomes
N: normal control
PPI: protein‐protein interaction

Cervical carcinoma is one of the leading causes of cancer‐related mortality in the world and accounts for 10–15% of tumor‐related deaths in women 1. Recently, the occurrence rate of cervical cancer is higher in younger women 2. Because of the progression in screening methods, more and more patients are diagnosed with cervical cancer at the early stage 3. Cervical intraepithelial neoplasia (CIN) is a class of precancerous lesions of cervical squamous cell carcinoma (CSCC). CIN can be categorized as CIN I, CIN II or CIN III. The risk of CIN I, CIN II and CIN II developing into cervical cancer was 1%, 5% and 12–22%, respectively. The possibility of CIN evolving into cervical carcinoma is 20‐fold higher than that of a normal cervix 4. Thus, the cure of CIN plays a key role in the prevention of cervical cancer. Up to now, the mechanism underlying the progression of CIN into CSCC remains poorly elucidated. Therefore, it is imperative to find diagnostic biomarkers that can contribute to exploring the mechanism in progression of CIN developing into CSCC.

miRNAs are a group of small, noncoding RNAs of 20–22 nucleotides that modulate about 60% of protein‐coding genes 5. miRNAs may play a key role in novel diagnostic, prognostic and therapeutic markers in clinical oncology. In addition, no curative therapy can be used for CSCC, and the current methods have only limited efficacy. Therefore, it is essential to identify accurate and credible biomarkers for CSCC diagnosis and treatment.

In our study, we intended to obtain more credible results than possible with individual study via integrated analysis. We performed functional annotation of differentially expressed genes (DEGs) and a CSCC‐specific miRNA‐target gene network to seek key DEGs and differentially expressed miRNAs (DEmiRNAs) in CIN and biomarkers in the development of CIN into CSCC.

## Materials and methods

### DEGs identification of CSCC and CIN

We searched gene expression datasets of CSCC and CIN from the Gene Expression Omnibus (GEO) database (http://www.ncbi.nlm.nih.gov/geo) 6. Search keywords were [‘cervical intraepithelial neoplasia’ (MeSH Terms) OR cervical intraepithelial neoplasia (All Fields)] OR {[‘cervical’ (MeSH Terms) OR cervical (All Fields)] AND [‘carcinoma’ (MeSH Terms) OR carcinoma (All Fields)]} AND ‘gse’ (Filter) 7. The study types were limited to ‘expression profiling by array’. Datasets that meet the following criteria would be included in our study: (a) the selected dataset must be genome‐wide mRNA or miRNA transcriptome data; (b) these data were obtained from tissues of the CIN, CSCC and normal control (N) (no drug stimulation or transfection); and (c) normalized or raw datasets were considered in this study. Finally, three datasets of mRNA and miRNA were screened and included. The study methodologies conformed to the standards set by the Declaration of Helsinki.

### Identification of DEGs and DEmiRNAs in CIN versus N and CIN versus CSCC

The method used for *P*‐value consolidation is the inverse normal method in the limma package and metaMA 8. The adopted standard is false discovery rate (FDR) < 0.05, and all datasets have the same direction of different expression. Finally, the DEGs and DEmiRNAs of CIN versus N and CIN versus CSCC were obtained. Intersection of DEGs and DEmiRNAs was obtained from CIN versus N and CSCC versus CIN.

### Gene ontology and pathway enrichment analysis of DEGs

To identify the characteristic biological function and potential pathways of common DEGs, we performed gene ontology (GO) and Kyoto Encyclopedia of Genes and Genomes (KEGG) pathway enrichment by using the online software genecodis3 9. All common DEGs were analyzed by GO and KEGG enrichment analysis using the r language (GSEABase package).

### **Protein**‐**protein interaction network construction of common DEGs**

Cytoscape 3.5.0 was used to search for all common DEGs based on the existing data of protein interaction in the String database. All common DEGs in CIN versus N and CIN versus CSCC groups were used to construct the protein‐protein interaction (PPI) network 10, 11, 12.

### DEmiRNA‐target interaction network in CIN versus N and CIN versus CSCC

miRTarBase (http://mirtarbase.mbc.nctu.edu.tw/php/index.php) 13 provides the latest and extensive experimentally validated miRNA‐target interaction information. The potential target genes of miRNAs were predicted by miRTarBase, which is an experimentally validated miRNA‐target interactions database in CIN versus N and CIN versus CSCC, respectively.

### Functional annotation of target mRNAs

Based on the earlier analysis results, we obtained seven target genes of DEmiRNAs. GO functional enrichment and KEGG functional enrichment analysis were performed on target genes using genecodis3 (http://genecodis.cnb.csic.es/analysis). FDR < 0.05 was defined as the criterion of statistical significance.

### Quantitative RT‐PCR confirmation

According to the results of GEO integrated analysis, we selected two DEmiRNAs (hsa‐miR‐99a and hsa‐miR‐21) and three targets including *TYMS*, *SASH1* and *Aurora kinase A* (*AURKA*) in CIN versus CSCC as candidate genes. A total of 12 blood samples were collected from four N subjects, four patients diagnosed with CIN and four patients diagnosed with CSCC. Informed written consent was obtained from all participants, and research protocols were approved by the Ethics Committee of our hospital.

## Results

### Differential expression analysis of genes in CIN and CSCC

After retrieving, we obtained three microarray studies of mRNA and three microarray studies of miRNA according to the inclusion criteria from the GEO database. The characteristics of the individual database for the integrated analysis are displayed in Table 1.

**Table 1:** mRNA and miRNA expression datasets used in this study.

GEO ID	Author	Platform	Samples (N : CIN : CESC)	Year
mRNA				
GSE63514	den Boon J	GPL570 [HG‐U133_Plus_2] Affymetrix Human Genome U133 Plus 2.0 Array	24 : 76 : 28	2015
GSE51993	Mo W	GPL10558 Illumina Human HT‐12 V4.0 expression bead chip	7 : 17 : 0	2013
GSE7803	Kuick R	GPL96 [HG‐U133A] Affymetrix Human Genome U133A Array	10 : 7 : 21	2007
miRNA				
GSE30656	Sie D	GPL6955 Agilent‐016436 Human miRNA Microarray 1.0 (Feature Number version)	10 : 18 : 10	2012
GSE46172	Mo W	GPL8179 Illumina Human v2 MicroRNA expression bead chip	7 : 17 : 0	2013
GSE19611	Pereira PM	GPL7534 National DNA microarray facility of University of Aveiro miRNA chip v1.1	23 : 16 : 4	2010

**Figure 1:**
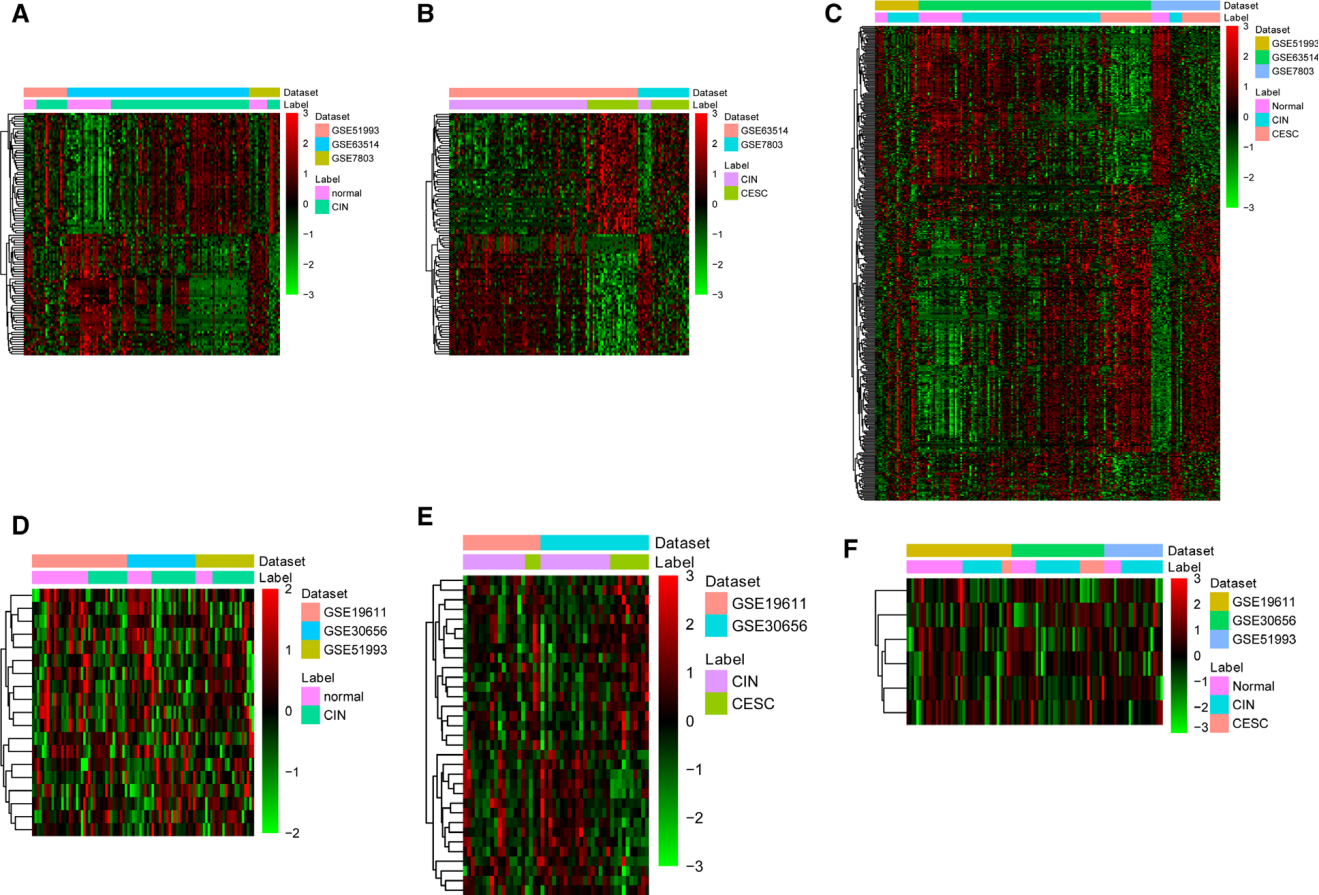
Heatmap image showing the DEGs that were significantly up‐regulated or down‐regulated (FDR < 0.05) in CIN versus N and CIN versus CSCC. (A) The top 100 most significantly up‐regulated and down‐regulated genes in CIN versus N. (B) The top 100 most significantly up‐regulated and down‐regulated genes in CSCC versus CIN. (C) Common DEGs in CIN versus N and CSCC versus CIN. (D) The top 100 most significantly up‐regulated and down‐regulated miRNAs in CIN versus N. (E) The top 100 most significantly up‐regulated and down‐regulated miRNAs in CSCC versus CIN. (F) Common DEmiRNAs in CIN versus N and CSCC versus CIN.

A total of 1375 DEGs were obtained with FDR < 0.05 in CIN compared with N, among which the expression level of 719 genes was increased and the expression level of 656 genes was decreased. Likewise, 2235 DEGs with 1213 up‐regulated and 1022 down‐regulated genes were obtained in CSCC compared with CIN. The top 20 DEGs in CIN versus N and CESC versus CIN are listed in Tables 2 and 3, respectively. The hierarchical clustering heatmap of the top 100 most significantly up‐regulated or down‐regulated genes in CIN versus N and CSCC versus CIN is shown in Fig. 1A,B. In total, 392 common DEGs were obtained by taking the intersection of CIN versus N and CSCC versus CIN (Fig. 1C).

**Table 2:** Most differentially expressed mRNAs in CIN versus N. Combined.ES, Combined Effect Size.

ID	Symbol	Combined.ES	*P*‐value	FDR	Up‐regulated or down‐regulated
4175	*MCM6*	1.63E+00	6.13E−14	7.02E−10	Up
6491	*STIL*	1.58E+00	1.24E−13	7.02E−10	Up
51514	*DTL*	1.61E+00	1.72E−13	7.02E−10	Up
2237	*FEN1*	1.52E+00	5.16E−13	1.58E−9	Up
8317	CDC7	1.58E+00	6.88E−13	1.68E−9	Up
4751	*NEK2*	1.50E+00	8.36E−13	1.71E−9	Up
5985	*RFC5*	1.49E+00	1.24E−12	2.17E−9	Up
1029	*CDKN2A*	1.50E+00	1.42E−12	2.17E−9	Up
10635	*RAD51AP1*	1.49E+00	2.18E−12	2.97E−9	Up
79022	*TMEM106C*	1.44E+00	3.50E−12	3.92E−9	Up
10493	*VAT1*	−1.31E+00	4.05E−10	1.15E−7	Down
2012	*EMP1*	−1.30E+00	5.22E−10	1.42E−7	Down
794	*CALB2*	−1.26E+00	1.10E−9	2.63E−7	Down
51754	*TMEM8B*	−1.24E+00	1.53E−9	3.38E−7	Down
79919	*C2orf54*	−1.23E+00	5.66E−9	8.77E−7	Down
23136	*EPB41L3*	−1.17E+00	7.59E−9	1.12E−6	Down
9846	*GAB2*	−1.17E+00	9.56E−9	1.36E−6	Down
10749	*KIF1C*	−1.15E+00	1.85E−8	2.36E−6	Down
5630	*PRPH*	−1.11E+00	3.33E−8	3.74E−6	Down
9778	*KIAA0232*	−1.13E+00	3.49E−8	3.85E−6	Down

**Table 3:** Most differentially expressed mRNAs in CESC versus CIN. Combined.ES, Combined Effect Size.

ID	Symbol	Combined.ES	*P*‐value	FDR	Up‐regulated or down‐regulated
7130	*TNFAIP6*	1.81E+00	8.88E−16	2.72E−12	Up
26585	*GREM1*	1.70E+00	3.57E−14	5.47E−11	Up
23350	*U2SURP*	1.67E+00	1.90E−13	2.12E−10	Up
10299	*6‐Mar*	1.57E+00	5.98E−13	4.88E−10	Up
6574	*SLC20A1*	1.55E+00	7.90E−13	6.04E−10	Up
87	*ACTN1*	1.46E+00	1.00E−11	5.12E−9	Up
5352	*PLOD2*	1.49E+00	1.20E−11	5.85E−9	Up
10721	*POLQ*	1.49E+00	3.02E−11	1.06E−8	Up
3836	*KPNA1*	1.40E+00	4.24E−11	1.27E−8	Up
9532	*BAG2*	1.44E+00	4.67E−11	1.36E−8	Up
8857	*FCGBP*	−2.26E+00	0	0	Down
6338	*SCNN1B*	−1.96E+00	0	0	Down
49860	*CRNN*	−1.81E+00	6.66E−16	2.72E−12	Down
4013	*VWA5A*	−1.74E+00	8.22E−15	2.01E−11	Down
22802	*CLCA4*	−1.70E+00	1.64E−14	3.03E−11	Down
11005	*SPINK5*	−1.69E+00	1.73E−14	3.03E−11	Down
7263	*TST*	−1.65E+00	5.26E−14	6.50E−11	Down
6947	*TCN1*	−1.68E+00	5.31E−14	6.50E−11	Down
51090	*PLLP*	−1.60E+00	2.09E−13	2.13E−10	Down
6590	*SLPI*	−1.59E+00	2.73E−13	2.57E−10	Down

Nineteen DEmiRNAs (7 up‐regulated DEmiRNAs and 12 down‐regulated DEmiRNAs) were obtained with *P* < 0.05 in CIN compared with N. Likewise, 33 DEmiRNAs with 18 up‐regulated and 15 down‐regulated genes were obtained in CSCC compared with CIN. The top 20 DEmiRNAs in CIN versus N and CESC versus CIN are listed in Tables 4 and 5, respectively. The hierarchical clustering heatmap of DEmiRNAs is shown in Fig. 1D,E. A total of six common DEmiRNAs were obtained by taking the intersection of CIN versus N and CSCC versus CIN (Fig. 1F).

**Table 4:** DEmiRNAs in CIN versus N. Combined.ES, Combined Effect Size.

Symbol	Combined.ES	*P*‐value	FDR	Up‐regulated or down‐regulated
hsa‐miR‐146a	7.82E−1	6.53E−4	2.84E−2	Up
hsa‐miR‐10a	7.63E−1	7.03E−4	2.84E−2	Up
hsa‐miR‐34b	6.76E−1	2.69E−3	3.83E−2	Up
hsa‐miR‐135b	6.26E−1	6.00E−3	6.06E−2	Up
hsa‐let‐7g	4.74E−1	3.71E−2	2.01E−1	Up
hsa‐miR‐25	4.54E−1	3.75E−2	2.01E−1	Up
hsa‐miR‐21	4.69E−1	4.10E−2	2.01E−1	Up
hsa‐miR‐203	−1.25E+00	3.35E−7	4.05E−5	Down
hsa‐miR‐149	−7.73E−1	1.60E−3	3.51E−2	Down
hsa‐miR‐210	−7.00E−1	2.88E−3	3.83E−2	Down
hsa‐miR‐23b	−6.61E−1	3.17E−3	3.83E−2	Down
hsa‐miR‐324‐3p	−6.14E−1	6.01E−3	6.06E−2	Down
hsa‐miR‐222	−5.88E−1	8.09E−3	7.33E−2	Down
hsa‐miR‐205	−5.47E−1	1.25E−2	9.81E−2	Down
hsa‐miR‐99a	−5.41E−1	1.40E−2	9.97E−2	Down
hsa‐miR‐24	−4.98E−1	2.24E−2	1.51E−1	Down
hsa‐miR‐30d	−4.75E−1	2.96E−2	1.88E−1	Down
hsa‐miR‐519d	−4.58E−1	3.83E−2	2.01E−1	Down
hsa‐miR‐214	−4.27E−1	5.00E−2	2.24E−1	Down

**Table 5:** DEmiRNAs in CESC versus CIN. Combined.ES, Combined Effect Size.

Symbol	Combined.ES	*P*‐value	FDR	Up‐regulated or down‐regulated
hsa‐miR‐16	1.15E+00	6.06E−4	2.31E−2	Up
hsa‐miR‐18a	1.13E+00	7.63E−4	2.31E−2	Up
hsa‐miR‐106a	1.01E+00	2.51E−3	4.34E−2	Up
hsa‐miR‐185	9.49E−1	3.84E−3	4.65E−2	Up
hsa‐miR‐200c	9.20E−1	5.42E−3	5.97E−2	Up
hsa‐miR‐31	8.72E−1	7.42E−3	7.45E−2	Up
hsa‐miR‐93	9.02E−1	8.00E−3	7.45E−2	Up
hsa‐miR‐106b	8.24E−1	1.09E−2	8.07E−2	Up
hsa‐miR‐9	8.06E−1	1.25E−2	8.07E−2	Up
hsa‐miR‐205	8.52E−1	1.38E−2	8.07E−2	Up
hsa‐miR‐125b	−1.52E+00	2.43E−5	2.94E−3	Down
hsa‐miR‐195	−1.31E+00	1.85E−4	1.12E−2	Down
hsa‐miR‐29a	−1.08E+00	1.42E−3	3.24E−2	Down
hsa‐miR‐99a	−1.05E+00	1.60E−3	3.24E−2	Down
hsa‐miR‐497	−9.61E−1	3.43E−3	4.65E−2	Down
hsa‐miR‐29c	−8.50E−1	8.82E−3	7.63E−2	Down
hsa‐miR‐100	−8.24E−1	1.10E−2	8.07E−2	Down
hsa‐let‐7c	−8.02E−1	1.32E−2	8.07E−2	Down
hsa‐miR‐145	−8.00E−1	1.36E−2	8.07E−2	Down
hsa‐miR‐26a	−7.79E−1	1.61E−2	8.10E−2	Down

### Functional annotation of common DEGs

**Figure 2:**
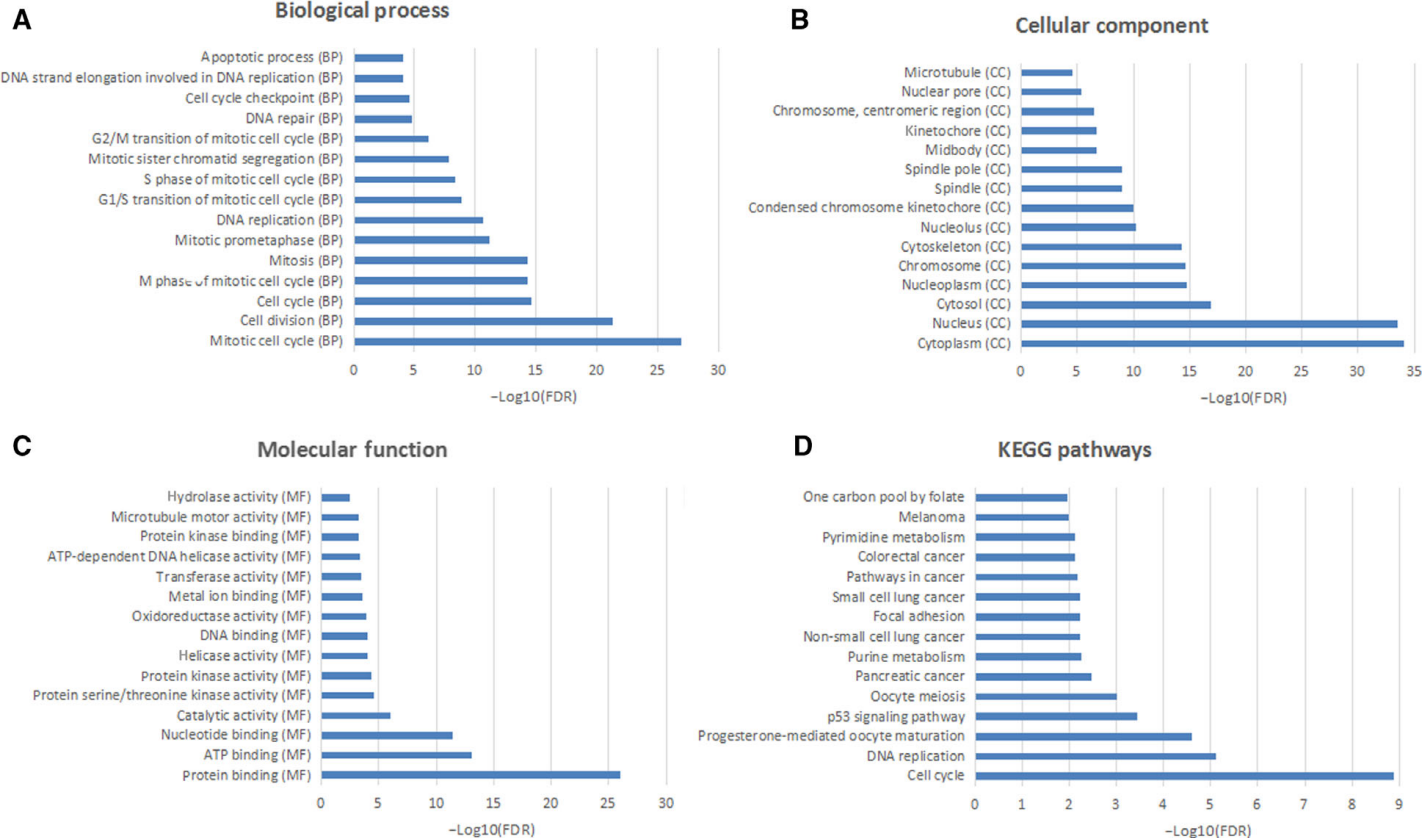
The top 15 most significantly enriched GO terms and KEGG pathways of common DEGs in CIN versus N and CSCC versus CIN. (A) The top 15 most significantly enriched GO terms of the biological process. (B) The top 15 most significantly enriched GO terms of the cellular component. (C) The top 15 most significantly enriched GO terms of molecular function. (D) The top 15 most significantly enriched KEGG pathways.

Functional annotation analysis manifested that these common DEGs were significantly involved in the mitotic cell cycle (FDR = 1.21E−27), cell division (FDR = 5.43E−22), protein binding (FDR = 9.90E−27), ATP binding (FDR = 7.68E−14), p53 signaling pathway (FDR = 0.0003714), cell cycle (FDR = 1.31E−9) and DNA replication (FDR = 7.79E−6) (Fig. 2). Details of all GO and KEGG items are listed in Table S1.

### PPI network and module analysis of common DEGs

**Figure 3:**
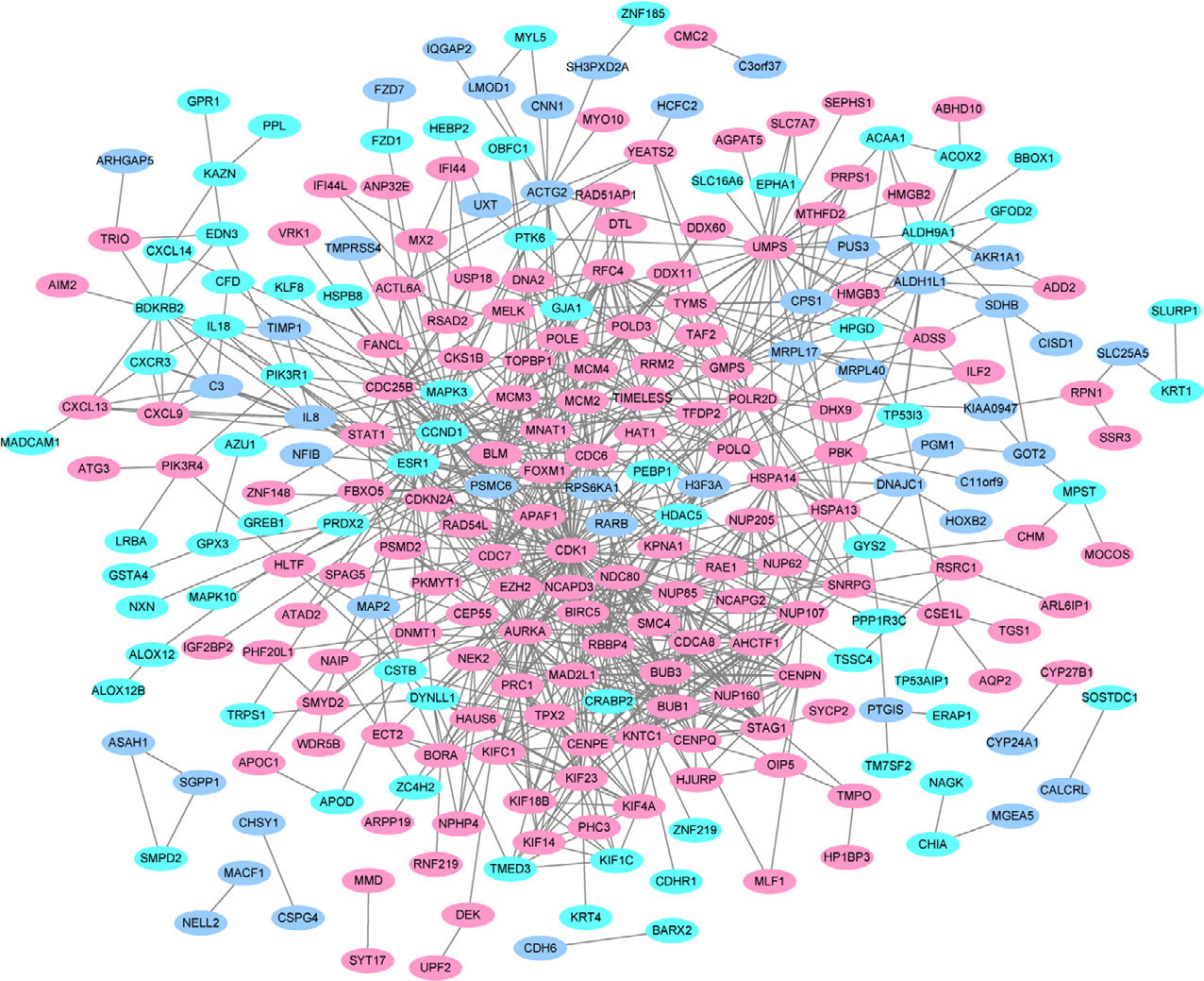
PPI network of common DEGs. Pink and aqua ellipses represent proteins encoded by up‐regulated and down‐regulated common DEGs, respectively. Purple ellipses indicate proteins encoded by common DEGs that have inconsistent expression levels in CIN versus N and CSCC versus CIN.

**Figure 4:**
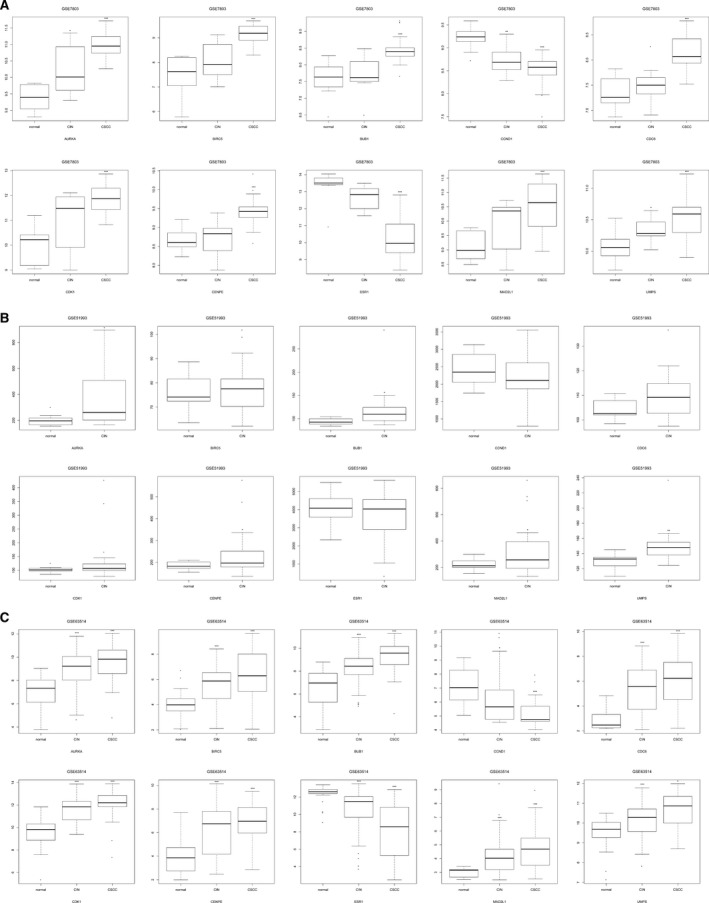
The expression level of hub genes (*CDK1*, *CCND1*, *ESR1*, *AURKA*, *BIRC5*, *MAD2L1*, *BUB1*, *CDC6*, *CENPE* and *UMPS*) in (A) GSE7803, (B) GSE51993 and (C) GSE63514. *P < 0.05, **P < 0.01, ***P < 0.001; the statistical *t*‐test was used to determine significance.

To identify potential interactions between common DEGs, we constructed a PPI network. In total, 259 nodes (genes) and 886 edges were identified among DEGs in the results, which are shown in Fig. 3. Among them, the higher degree genes are *CDK1* (degree = 70), *CCND1* (degree = 32), *ESR1* (degree = 31), *AURKA* (degree = 30), *BIRC5* (degree = 30), *MAD2L1* (degree = 28), *BUB1* (degree = 26), *CDC6* (degree = 26), *CENPE* (degree = 26) and *UMPS* (degree = 26). The expression levels of these genes in three databases are shown in Fig. 4.

### DEmiRNA‐target interaction network in CIN versus N and CIN versus CSCC

**Figure 5:**
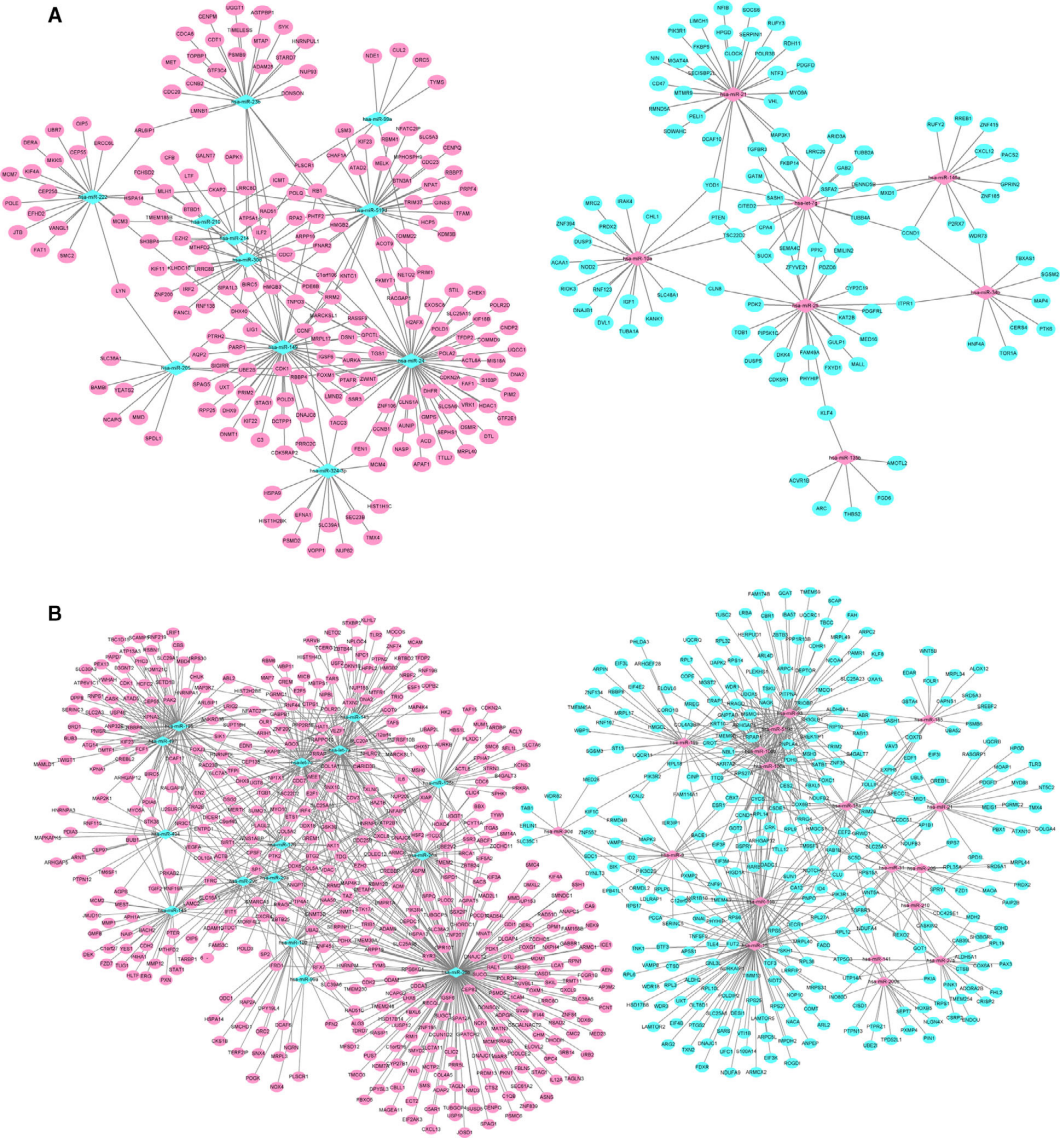
DEmiRNA‐mRNA interaction network. Green represents down‐regulation of miRNAs or targets; red represents the up‐regulation of miRNAs or targets. (A) CIN versus N. (B) CSCC versus CIN.

In total, 393 DEmiRNA‐target interaction pairs were obtained. The DEmiRNA‐target regulatory network was constructed based on these DEmiRNA‐target interaction pairs, which consisted of 326 nodes and 393 edges (Fig. 5A). Based on the CIN‐specific DEmiRNA‐target interaction network, has‐miR‐24 (degree = 64), has‐miR‐149 (degree = 45) and has‐miR‐519d (degree = 38) were the top three DEmiRNAs that regulated most DEGs. All of these DEmiRNAs were down‐regulated in CIN based on the GEO database.

**Figure 6:**
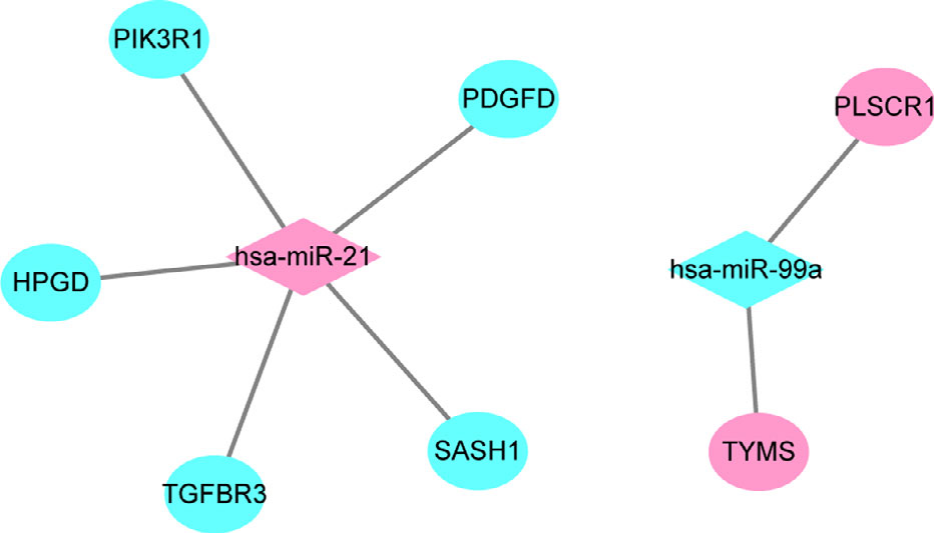
Seven DEmiRNA‐mRNA interaction pairs that were intersected in both CIN versus N and CSCC versus CIN.

Likewise, 1396 DEmiRNA‐target interaction pairs were obtained, which consisted of 828 nodes and 1396 edges (Fig. 5B). Based on the CIN‐specific DEmiRNA‐target interaction network, has‐miR‐26b (degree = 212), has‐miR‐16 (degree = 98) and has‐let‐7a (degree = 82) were the top three DEmiRNAs. All of these DEmiRNAs were down‐regulated in CIN based on the GEO database. Also, the intersection of seven DEmiRNA‐target pairs was obtained in the above two sections. Among them, two interaction pairs (hsa‐miR‐21‐SASH1 and hsa‐miR‐99a‐TYMS) were involved in CIN and the progression of CIN into CSCC (Fig. 6). Topological properties of the DEmiRNA‐mRNA interaction pair of CIN versus N and CESC versus CIN are shown in Tables S2 and S3, respectively. 

### Functional annotation of DEmiRNA targets

**Figure 7:**
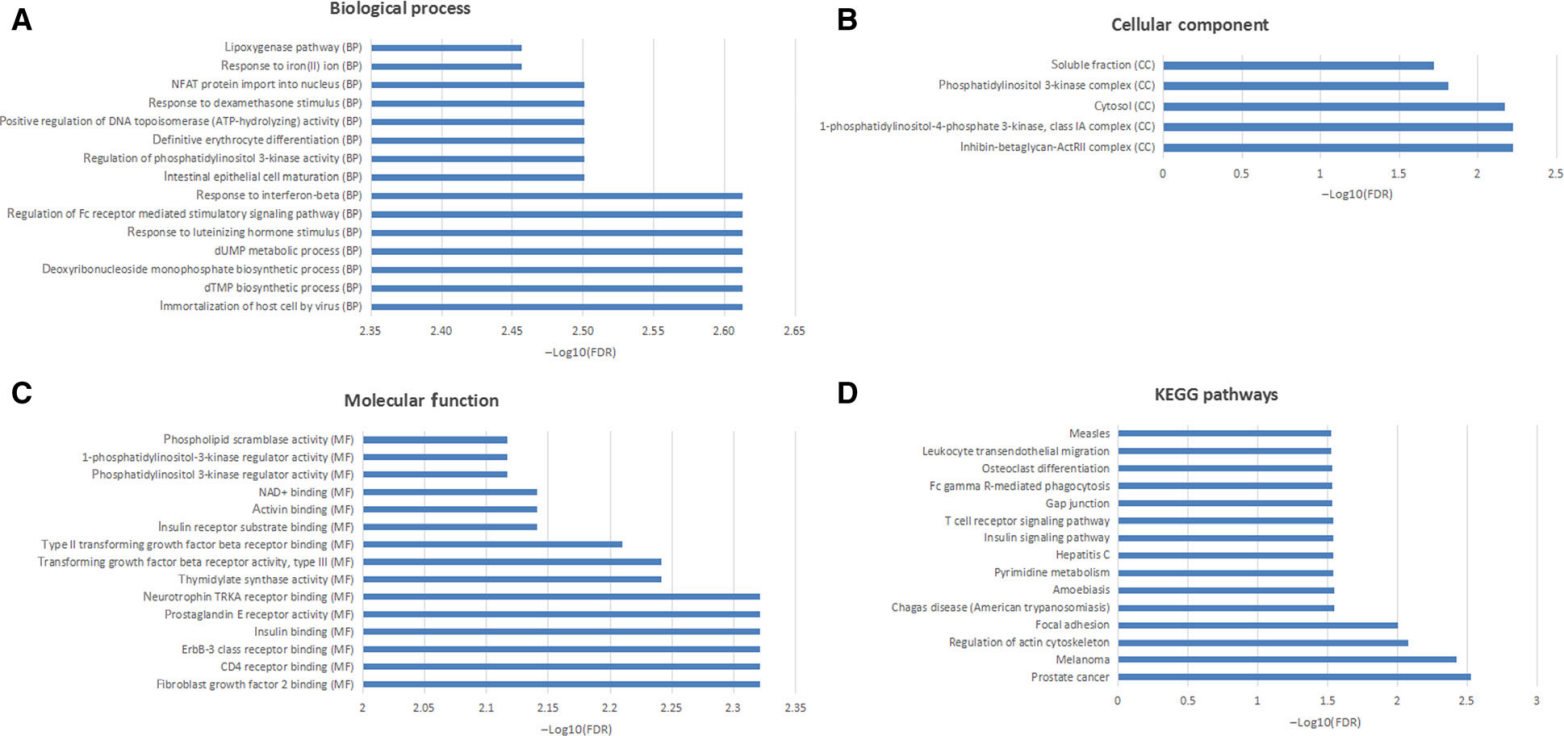
The top 15 most significantly enriched GO terms and KEGG pathways of DEmiRNAs targets. (A) The top 15 most significantly enriched GO terms of the biological process. (B) The top 15 most significantly enriched GO terms of the cellular component. (C) The top 15 most significantly enriched GO terms of molecular function. (D) The top 15 most significantly enriched KEGG pathways.

Figure 7, immortalization of host cell by virus (FDR = 0.00243696), dTMP biosynthetic process (FDR = 0.00243696), deoxyribonucleoside monophosphate biosynthetic process (FDR = 0.00243696), inhibin‐betaglycan‐ActRII complex (FDR = 0.00593324), fibroblast growth factor 2 binding (FDR = 0.00477304) and CD4 receptor binding (FDR = 0.00477304) were the most significantly enriched GO terms. Prostate cancer (FDR = 3.00E−3) and melanoma (FDR = 3.00E−3) were two significantly enriched pathways. Details of all GO and KEGG items are listed in Table S4, among which pathway enrichment analysis for *TYMS*, *CDK1*, *AURKA* and *SASH1* was further performed in SMPDB, BIOCYC, KEGG and REACTOME databases, which is shown in Table 6.

**Table 6:** Pathway enrichment analysis for four DEGs in SMPDB, BIOCYC, KEGG and REACTOME databases. Combined.ES, Combined Effect Size.

Database	Gene name	Pathway
SMPDB	*TYMS*	beta‐Ureidopropionase deficiency Dihydropyrimidinase deficiency MNGIE (mitochondrial neurogastrointestinal encephalopathy) UMP synthase deficiency (orotic aciduria)
	*CDK1*	–
	*AURKA*	–
	*SASH1*	–
BIOCYC	*TYMS*	dTMP de novo biosynthesis (mitochondrial) Pyrimidine deoxyribonucleotides salvage Pyrimidine deoxyribonucleotides de novo biosynthesis Pyrimidine deoxyribonucleotides biosynthesis from CTP
	*CDK1*	–
	*AURKA*	–
	*SASH1*	–
KEGG	*TYMS*	Thymidylate synthase
	*CDK1*	Cyclin‐dependent kinase 1
	*AURKA*	Aurora kinase A
	*SASH1*	SAM and SH3 domain containing 1
REACTOME	*TYMS*	Cell cycle (*Homo sapiens*)
	*CDK1*	Cell cycle (*Homo sapiens*)
	*AURKA*	Gene expression (transcription) (*Homo sapiens*), metabolism of proteins (*Homo sapiens*)
	*SASH1*	–

### Quantitative RT‐PCR confirmation

**Figure 8:**
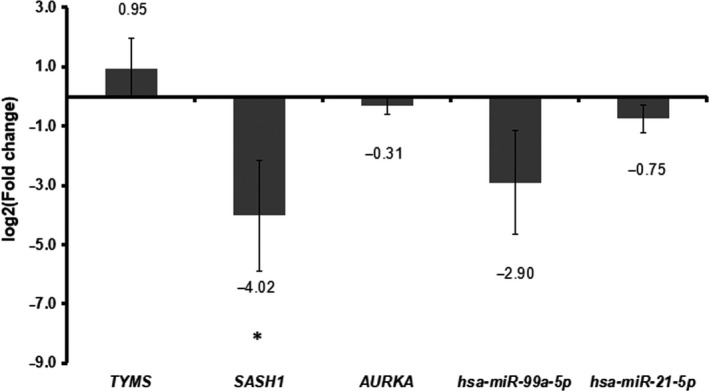
Quantitative RT‐PCR results of three DEGs (*TYMS*, *SASH1* and *AURKA*) and two DEmiRNAs (hsa‐miR‐21 and hsa‐miR‐99a) in CIN versus N. The error bars indicate SD. The statistical test used to determine significance was one‐way ANOVA. All of the data had biological duplicates, and each gene had three duplicates. *SASH1 has significant difference.

To indicate the results of integrated analysis, we selected two DEmiRNAs (hsa‐miR‐99a and hsa‐miR‐21) and three target genes including *TYMS*, *SASH1* and *AURKA* in CIN versus CSCC. Based on the results of quantitative RT‐PCR, the expression of hsa‐miR‐99a, hsa‐miR‐21, *AURKA* and *SASH1* was down‐regulated, whereas the expression of *TYMS* was up‐regulated in CIN compared with CSCC. The expression of *TYMS*, *SASH1* and hsa‐miR‐99a was consistent with the results of our integrated analysis (Fig. 8).

## Discussion

CIN is a class of precancerous lesions of the CSCC, but the mechanisms of CIN developing into CSCC need to be elucidated more clearly 2. In this study, we obtained 1375 DEGs and 19 DEmiRNAs in tissues of patients with CIN compared with N. Likewise, there were 2235 DEGs and 33 DEmiRNAs in tissues of patients with CSCC compared with patients with CIN. A total of four DEGs (*AURKA*, *SASH1*, *CDK1* and *TYMS*) under the regulation of two DEmiRNAs (hsa‐miR‐21 and hsa‐miR‐99a) were associated with CIN and CSCC.

*CDK1* and *AURKA* are two major hallmarks of both CIN versus N and CIN versus CSCC. *CDK1* has more than 70 regulatory targets, which play a vital role in the control of the cell cycle. In transcription and cell‐cycle progression, various target substrates are directly phosphorylated by *CDK1* in response to various stimuli 14. As previously described, many patients with cancers had aberrant activation of CDKs and their modulators. Abnormal cell proliferation and genomic instability were caused by dysregulation of CDKs 15. According to our integrated analysis, up‐regulated *CDK1* was modulated by hsa‐miR‐205 and hsa‐miR‐24 in the tissues of CIN versus N and by hsa‐miR‐195 and hsa‐miR‐497 in CIN versus CSCC, which have the same pattern in a previous study 16. In the PPI analysis, *CDK1* has the highest degree among the hub proteins. Also, *CDK1* was enriched in the p53 signaling pathway in the KEGG analysis. Luo *et al*. 16 reported that *CDK1* played a complicated role in regulating genetic networks involved in the progression of cervical cancer. Prognosis of advanced stage cervical cancer may be enhanced by new therapeutics targeting *CDK1* or its related pathways. This was consistent with our results, which indicated that *CDK1* might serve as a biomarker for CIN and the progression of CIN into CSCC.

*AURKA* is a member of a family of mitotic serine/threonine kinases. During mitosis and meiosis, *AURKA *is correlated with crucial processes, whose appropriate function is integral for normal cell proliferation 17. The first study related to the family of kinases in tumorigenesis reported that Aurora A and B were overexpressed in primary breast and colon tumor samples. Emerging studies found that *AURKA* was amplified or overexpressed in other tumor types, such as pancreatic, ovarian and hepatocellular tumors 18, 19, 20. Nae‐Fang Twu *et al*. 21 have shown that expression of *AURKA *was significantly increased in carcinoma and CIN 3 compared with the normal cervix. In our study, *AURKA* was up‐regulated in CIN versus N and CIN versus CSCC, which showed the same pattern with the previous study 21. In the PPI analysis, AURKA was among the top four hub proteins. In the GO and KEGG analysis, *AURKA* was enriched in the items of mitotic cell cycle, cell division and oocyte meiosis.

Recently, many studies reported that *SASH1* played a crucial role in inhibiting various tumors. For example, He *et al*. 22 reported that the gene of *SASH1 *inhibited the metastatic progression of hepatocarcinoma cells via regulating the sonic hedgehog signaling pathway. Ren *et al*. 23 showed that up‐regulation of *SASH1* inhibited proliferation and migration of ovarian carcinoma cells. In line with these studies, another study 24 showed that *SASH1* was down‐regulated in cervical cancer, indicating that* SASH1 *might play a negative role in cervical cancer. Up‐regulated *TYMS* by hsa‐miR‐99a and down‐regulated *SASH1 *by hsa‐miR‐21 were detected in CIN versus N and CIN versus CSCC in our study, indicating its important role in CIN, and up‐regulated *TYMS* and down‐regulated *SASH1* might serve as a biomarker for CIN and the progression of CIN into CSCC. In addition, the expression of these two DEmRNAs in CIN in our integrated analysis was consistent with our quantitative RT‐PCR results.

Recently, miRNAs have been described as potential diagnostic or prognostic markers for many cancers and can function as neoteric targets for cancer therapies, including cervical cancer 25, 26. As previously reported, miR‐21‐5p was expressed abnormally in patients with CSCC 27. Likewise, other researchers have reported that miR‐21 influenced tumorigenesis in the cervical squamous cell and served as an oncology miRNA (oncomiRNA) in cervical cancer 28, 29, 30. Up‐regulated hsa‐miR‐21 was detected in our integrated analysis. There were 50 and 34 targets of hsa‐miR‐21 in the DEmiRNA‐target interaction network in CIN versus N and CIN versus CSCC, respectively.

Many studies have indicated that miR‐99a is correlated with tumor pathogen 31, 32, 33. The miR‐99a is down‐regulated in human cancers, such as endometrioid endometrial carcinoma, suggesting that miR‐99a may inhibit tumor progression 34. However, the role of miR‐99a in cervical cancer still needs to be elucidated. A previous study provided evidence that miR‐99a was down‐regulated in cervical cancer tissues 35. Our results revealed that hsa‐miR‐99a was down‐regulated in CIN versus N and CIN versus CSCC. There were 7 and 12 targets of hsa‐miR‐99a in the DEmiRNA‐target interaction network in CIN versus N and CIN versus CSCC, respectively.

In conclusion, four DEGs (*TYMS*, *SASH1*, *CDK1* and *AURKA*) and two DEmiRNAs (hsa‐miR‐21 and hsa‐miR‐99a) may be involved in the pathogenesis of CIN and the progression of CIN into CSCC, which might contribute to developing novel diagnostic and therapeutic strategies for early‐stage CIN. Among them, *TYMS *was regulated by hsa‐miR‐99a and *SASH1* was regulated by hsa‐miR‐21.

## Conflict of interest

The authors declare no conflict of interest.

## Author contributions

DS and LH contributed to the conception of the study. RC and HW contributed the materials and performed the experiment. JJ and YD performed the data analyses. YD and XY contributed significantly in writing the manuscript. All authors read and approved the final manuscript.

## Supporting information


**Table S1** **. **GO (biological process, cellular component and molecular function) and KEGG items of common DEGs.

**Table S2**. Topological properties of the DEmiRNA‐mRNA interaction pair of CIN versus N.

**Table S3**. Topological properties of the DEmiRNA‐mRNA interaction pair of CESC versus CIN.

**Table S4** **. **GO (biological process, cellular component and molecular function) and KEGG items of DEmiRNA targets.
